# Supplementary material for: Complexome profiling of the Chlamydomonas psb28 mutant reveals TEF5 as an early PSII assembly factor
Source: Plant Cell. 2025 Mar 17;37(6):koaf055. doi: 10.1093/plcell/koaf055 (PMC12164590; doi:10.1093/plcell/koaf055)
Supplement: koaf055_Supplementary_Data [file koaf055_supplementary_data.zip › psb28_interactive.zip!/plots/intensity/qualityPlots/peptides/ZDS1_Cre07.g314150.t1.2.html]

### Cre07.g314150.t1.2

Trivial name: ZDS1  
SeqCoverage [median]: 29.90  
Mass: 63.41 kDa  
  
 

| Sequence | MissedCleavage | Mass [Da] | wtIntensity | muIntensity | Score |
| --- | --- | --- | --- | --- | --- |
| AFFTTPQLEPVDK | 0 | 1491.76 | 1121.06 | 50032.91 | 128.81 |
| AQASDVNAVGLK | 0 | 1171.62 | 2250.36 | 1489.90 | 107.21 |
| AQASDVNAVGLKDVPLR | 1 | 1751.95 | 3153.15 | 15434.68 | 87.39 |
| EFDNIYK | 0 | 927.43 | 0.00 | 986.56 | 75.31 |
| FYINEMK | 0 | 943.45 | 0.00 | 4081.03 | 133.81 |
| KAQASDVNAVGLKDVPLR | 2 | 1880.05 | 0.00 | 441.90 | 48.22 |
| LANSLALGTSPIVR | 0 | 1410.82 | 2876.51 | 18252.41 | 170.04 |
| LLAPIADYIK | 0 | 1115.66 | 8961.56 | 23802.14 | 87.31 |
| LVGVPVITVQLR | 0 | 1292.82 | 2145.93 | 0.00 | 87.31 |
| QDYIDSMEGATLSGR | 0 | 1641.73 | 1267.75 | 1134.06 | 129.54 |
| QLFPSAR | 0 | 817.44 | 0.00 | 837.34 | 89.37 |
| QYKEFDNIYK | 1 | 1346.65 | 0.00 | 1540.68 | 61.76 |
| SLFPDEPKPPAPGAPK | 1 | 1646.87 | 1907.82 | 5764.34 | 55.84 |
| SLTTAARPLAGASA | 1 | 1285.70 | 8037.62 | 20664.08 | 172.17 |
| SLVDPEGGMR | 0 | 1059.50 | 3794.01 | 6701.01 | 80.44 |
| SLVDPEGGMRDIR | 1 | 1443.71 | 1112.31 | 3135.75 | 71.24 |
| TDASVLR | 0 | 760.41 | 2050.71 | 7638.07 | 124.59 |
| VVTADAYVAALDVPGIK | 0 | 1700.93 | 1077.04 | 0.00 | 100.55 |
| VVTADAYVAALDVPGIKK | 1 | 1829.03 | 5022.77 | 13650.88 | 82.01 |
|  |  |  | 44778.6 | 175587.7 |  |
